# Reply to: Comment on: “The Benefits of Resistance Training in Obese Adolescents: A Systematic Review and Meta-analysis”

**DOI:** 10.1186/s40798-022-00547-3

**Published:** 2023-02-08

**Authors:** Bruno Ribeiro, Pedro Forte, Raquel Vinhas, Daniel A. Marinho, Luís B. Faíl, Ana Pereira, Fernando Vieira, Henrique P. Neiva

**Affiliations:** 1 Department of Sports Sciences, University of Beira Interior, 6200 Covilhã, Portugal; 2 Research Center in Sports Sciences, Health Sciences and Human Development, CIDESD, 6200-151 Covilhã, Portugal; 3 Instituto Politécnico de Bragança, Bragança, Portugal; 4 Higher Institute of Educational Sciences of the Douro, Penafiel, Portugal; 5 NOVA School of Science and Technology, Universidade NOVA de Lisbon, Lisbon, Portugal; 6 Department of Science and Technology, Polytechnic Institute of Setubal, 2910-761 Setúbal, Portugal; 7 KinesioLab – Research Unit in Human Movement, Piaget Institute, Lisbon, Portugal; 8 RECI – Research Unit in Education and Community Intervention, Piaget Institute, Lisbon, Portugal; 9 ISEIT de Almada, Piaget Instituto, Lisbon, Portugal

**Dear Editor,**

We thank Dr. Zhang [1] for his interest in our literature review about the effects of resistance training (RT) programs in obese adolescents [2]. In the review, we analyzed 21 studies to evaluate the impact of RT on body mass index, body fat, waist circumference, lean mass, insulin sensitivity, muscle strength, and cardiorespiratory fitness. Our main findings showed that RT programs seem to be positive for obese adolescents, improving muscle strength and cardiorespiratory fitness and reducing body fat, waist circumference, and body mass index. Yet, we were clear to advise the reader that the results should be carefully analyzed, and some limitations were addressed, for example, (i) the small number of participants in each study; (ii) the use/comparison of different training programs (i.e., varying durations, intensities, and exercises); (iii) methodological issues (i.e., with an unclear or high risk of bias); (iv) unclear dietary control of participants; and (v) maturational-related issues. We understand that these limitations should not refrain professionals from critically appreciating our results and then designing RT programs for obese adolescents. Dr. Zhang [1] lays out some specific methodological issues to facilitate subsequent studies, which we find a valuable contribution to the discussion and analysis of our findings. In our opinion, the details pointed out by Dr. Zhang [1] do not compromise any of the results and analysis provided, and any possible adjustment will not lead to changes in the main findings. Therefore, these comments should be seen as a complement to the analysis provided and some suggestions for future studies.

Despite the strategy of the search being clarified in the manuscript, some more details can be provided. Considerable effort was undertaken to make our search as wide as possible and to include as many results as we could before exclusion. Specifically, before the search, we made a word list based on keywords from the main research question and from primary searches in databases. Based on that, we decided to conduct the search in four databases, identifying original articles using the keywords: ("adolescence OR teenager") AND ("resistance training OR resistance exercise") AND ("obesity OR loss of weight"). The number of records that were subsequently identified was 5670, narrowed down to 2500 after the first screening. This is a large database, and we believe that it includes all relevant manuscripts published. Following Dr. Zhang’s suggestion, we decided to compare the use of the words “Adolescence” and “Adolescent” in our Boolean search and the results were the same. Perhaps this happens because the use of medical subject headings (MeSH) was not very common for a long period of time in sport sciences. However, the authors have been increasingly aware of the importance of their use to make indexing, cataloguing, and searching for articles more efficient and easier.

**Fig. 1:**
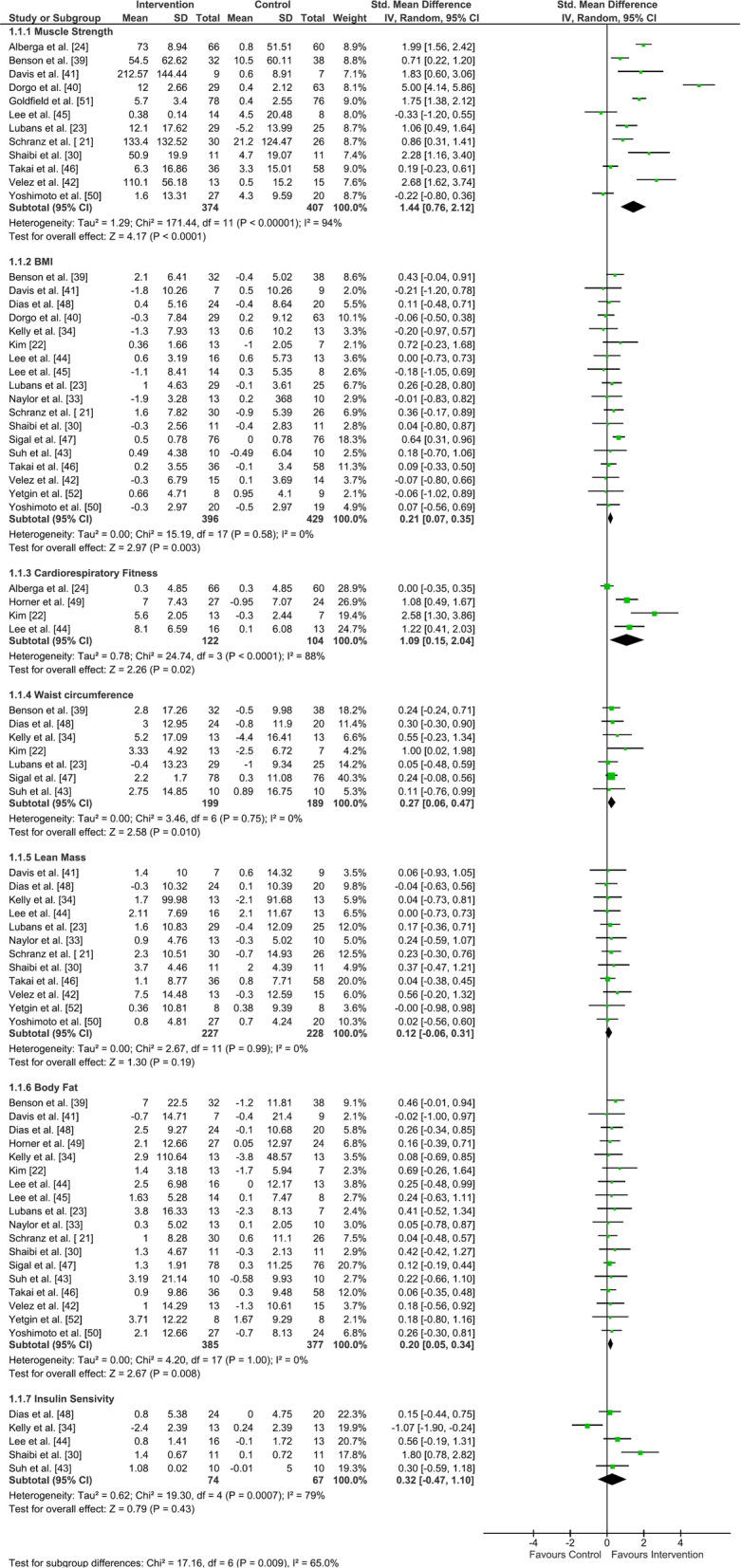
Forest plot of comparison for muscle strength, body mass index (BMI), cardiorespiratory fitness, waist circumference, lean mass, body fat, and insulin sensitivity. The center of each square represents the standard mean difference for individual trials, and the corresponding horizontal line represents the 95% confidence interval (CI). The diamonds represent pooled results

The second issue reported by Dr. Zhang [1] relates to the forest plot drawn [2]. Forest plots are commonly used to present information from individual studies, an estimate of the overall results, and a visual assessment of variation between the results of the studies. All these data are presented in Fig. 2 of our review [2]. Dr. Zhang [1] argued that there is an error in the forest plot, considering that muscle strength, body mass index, cardiorespiratory fitness, waist circumference, lean mass, body fat, and insulin sensitivity are not the same type of outcome. We agree with Dr. Zhang on this, and that is why our forest plot analysis was divided by each outcome. As far as we understand, the conflict emerged regarding the existence of an overall analysis that combined the weights of all studies. After correcting the approach according to Dr. Zhang’s suggestion (Fig. 1), we can verify that the effect size remained the same for each study and each outcome, and the overall effect and heterogeneity values were also the same. The only result that changed by using subtotals analysis only was the absolute weight value of each study. Nevertheless, the relative influence of each study remained the same. (The relative weight between studies was similar.) So, we might suggest this is not an error but rather a different way to present data results, without any implications for the meta-analysis results, discussion, conclusion, and practical applications presented.

In his final comment, Dr. Zhang [1] suggested including funnel plots analysis and using the Grading of Recommendations Assessment, Development, and Evaluation (GRADE) instrument. The funnel plot is a simple scatter plot commonly used to visually assess publication bias. During the process of data analysis, we designed the funnel plot for the included studies and each outcome, but we decided to continue only with the bias analysis presented in our review [2]. Some authors have argued that a visual interpretation of a funnel plot is too subjective to be useful and may give a misleading impression of publication bias [3, 4]. There has been some controversy in the literature about using this method in a meta-analysis, and this would only confuse the reader. Nevertheless, we can provide the funnel plot figure on request. Some issues regarding the use of grading systems are also reported by the literature [5, 6]. Among these, we cannot neglect the high level of subjectivity that comprises these grading systems and, ultimately, compromises reader interpretation. The consequences of inaccurate grading can be serious. For example, if the evidence is graded as low due to ineffective use of GRADE, professionals may conclude there is no need to use RT in obese adolescents when the literature is clear on this [2].

We hope that this response clarifies aspects that were pointed out, providing some more details about our review [2]. We believe that our review [2] summarizes the current state of research, highlighting the clearest effects found, but at the same time, being aware of the main limitations in included studies. Besides the main findings and practical suggestions, this review was intended to stimulate discussion and provide future directions for studying the influence and effects of RT, specifically, in obese adolescents.
